# Murine Toll-Like Receptor 2 Activation Induces Type I Interferon Responses from Endolysosomal Compartments

**DOI:** 10.1371/journal.pone.0010250

**Published:** 2010-04-20

**Authors:** Nicole Dietrich, Stefan Lienenklaus, Siegfried Weiss, Nelson O. Gekara

**Affiliations:** Molecular Immunology, Helmholtz Centre for Infection Research, Braunschweig, Germany; New York University, United States of America

## Abstract

**Background:**

Toll-like receptors (TLRs) are among the first-line sentinels for immune detection and responsiveness to pathogens. The TLR2 subfamily of TLRs (TLR1, TLR2, TLR6) form heterodimers with each other and are thus able to recognize a broad range of components from several microbes such as yeast, Gram-positive bacteria and protozoa. Until now, TLR2 activation by bacterial ligands has long been associated with pro-inflammatory cytokines but not type I interferon responses.

**Methodology/Principal Findings:**

Using a variety of transgenic mice, here we provide *in vivo* and *in vitro* data showing that TLR2 activation does in fact induce interferon-beta and that this occurs via MyD88-IRF1 and -IRF7 pathways. Interestingly, by microscopy we demonstrate that although a cell surface receptor, TLR2 dependent induction of type I interferons occurs in endolysosomal compartments where it is translocated to upon ligand engagement. Furthermore, we could show that blocking receptor internalization or endolysosomal acidification inhibits the ability of TLR2 to trigger the induction type I interferon but not pro-inflammatory responses.

**Conclusion/Significance:**

The results indicate that TLR2 activation induces pro-inflammatory and type I interferon responses from distinct subcellular sites: the plasma membrane and endolysosomal compartments respectively. Apart from identifying and characterizing a novel pathway for induction of type I interferons, the present study offers new insights into how TLR signaling discriminates and regulates the nature of responses to be elicited against extracellular and endocytosed microbes. These findings may also have clinical implication. Excessive production of pro-inflammatory cytokines and type I IFNs following activation of TLRs is a central pathologic event in several hyper-inflammatory conditions. The discovery that the induction of pro-inflammatory and type I IFN responses can be uncoupled through pharmacological manipulation of endolysosomal acidification suggests new avenues for potential therapeutic intervention against inflammations and sepsis.

## Introduction

Pattern recognition receptors (PRRs) serve as first-line sentinels for immune detection of pathogens. To date, the best characterized PRRs are the Toll-like receptors (TLRs) expressed either on the cell surface (TLR1, 2, 4, 5, 6 and 11) or on endosomal membranes (TLR3, 7, 8 and 9) [1]. Stimulation of TLRs triggers activation of MyD88-dependent signaling pathways as well as TRIF-dependent pathways that are unique to TLR3 and TLR4 [2], [3], [4]. These pathways activate various transcription factors like NF-κB, AP-1 and/or interferon regulatory factors (IRFs). This results in the transcription of genes encoding pro-inflammatory cytokines, chemokines and/or type I interferons (IFNs) that orchestrate anti-bacterial and anti-viral responses [2], [5], [6].

Although originally discovered based on their ability to interfere with viral replication, type I IFNs are also produced in response to bacterial and even parasitic infections [5], [7]. Type I IFNs encompass a family of more than 13 IFN-α isotypes, a single IFN-β isotype, and others, such as IFN-ω, -ε and -κ [5], [6], [8], [9]. They all signal through a common two-chain receptor (IFNAR) and activate a large set of IFN-inducible genes that exert widely overlapping pleiotropic immunomodulatory effects central to the activation of innate and adaptive immunity.

Of the PRRs, broadly, two categories of receptors are known to induce type I IFNs: the transmembrane TLRs and the cytosolic receptors [5], [8]. The latter include the RIG-I helicase and MDA5 which recognize cytosolic RNA and DNA [5], [8], [10], [11]

The TLR2 subfamily of receptors (TLR2/TLR1 and TLR2/TLR6 heterodimers) recognizes a broad range of cell surface components from yeast, Gram-positive bacteria, viruses and even protozoa [5], [12]. By virtue of their cell surface localization, in principle, these receptors are among the first to interact with and hence initiate immune reactions against such microbes. So far,these receptors have been assumed to elicit pro-inflammatory but not type I interferon responses in response to activation by bacterial ligands, [13], [14], [15], [16], [17], [18]. We now show that ligands for TLR2/1 and TLR2/6 heterodimers do induce type I IFN responses. Although a cell surface receptor, we observed that upon activation, TLR2 is internalized and transported into endolysosomal compartments from where it induces interferon-β via MyD88-IRF1 and IRF7 dependent pathways. Consequently, inhibition of receptor internalization or endosomal acidification could block the induction of IFN-β and IFN-inducible genes but not pro-inflammatory cytokines like TNF-α. This suggests that TLR2 activation induces pro-inflammatory and type I interferon responses from distinct sub-cellular sites: the plasma membrane and endolysosomal compartments, respectively. The spatio-temporal diversification of TLR2 signaling offers new insights into how TLR signaling discriminates and regulates the nature of responses to be elicited against extracellular and endocytosed microbes.

## Results

### TLR2 induces type I IFN responses

**Figure 1:**
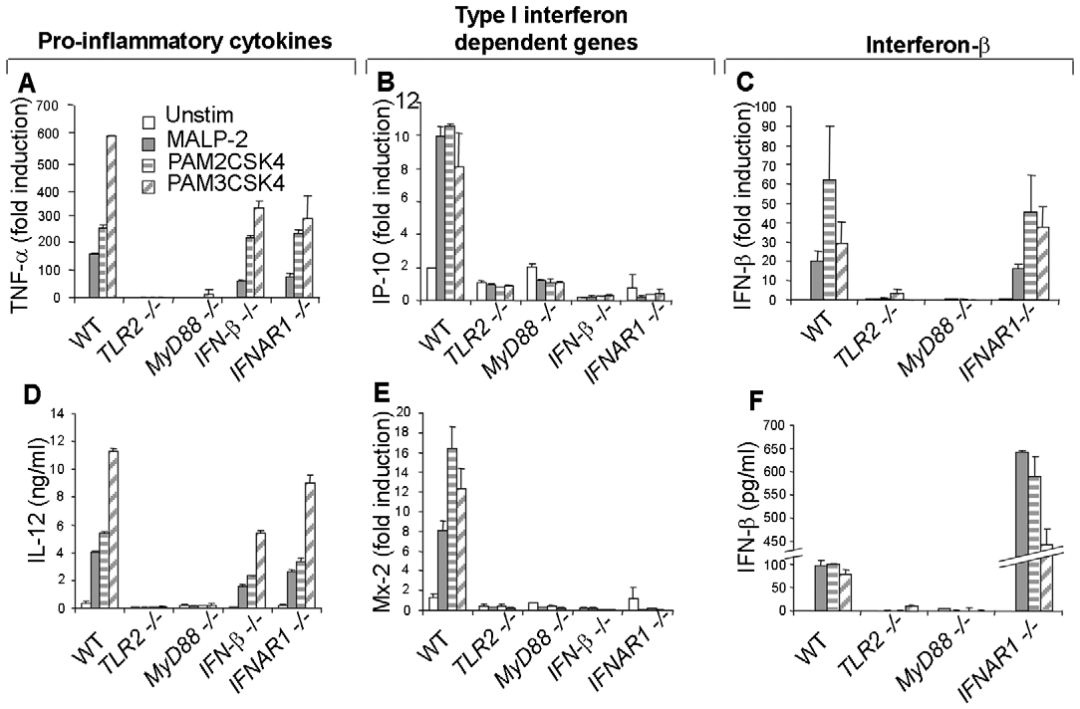
TLR2 directly induces pro-inflammatory and type I IFN dependent responses via MyD88. WT, *TLR2^−/−^*, *MyD88^−/−^*, *IFN-β^−/−^* and *IFNAR1^−/−^* macrophages were stimulated with 1.5 µg/ml of MALP-2 or PAM2CSK4 or PAM3CSK4 then analyzed 6 h later by real-time RT-PCR for mRNAs of TNF-α (**A**), IP-10 (**B**), Mx-2 (**E**) and IFN-β (**C**). In parallel, culture supernatants of cells stimulated for 24 h or 10 h were analyzed by ELISA for concentrations of IL-12p70 (**D**) or IFN-β (**F**) respectively. Data are representative of at least three independent experiments (mean ± s.e.m).

TLR2 activation by bacterial ligands is generally assumed to induce the transcription of canonical NF-κB dependent pro-inflammatory cytokines but not type I interferons (IFNs) or interferon-inducible genes [13], [14], [15], [16], [17]. However, when we stimulated bone marrow derived macrophages (BMDMs) we noted that besides pro-inflammatory cytokines like TNF-α and IL-12 (**Fig. 1A and D**), ligands for TLR2/TLR1 (PAM3CSK4) and TLR2/TLR6 (MALP-2, PAM2CSK4), could also trigger the induction of type I IFN-inducible genes; IP-10 (CXCL-10), MX-2, IL-6 and iNOS (for NO production) (**Fig. 1B, E** and **Fig. S1**).

An initial interpretation of type I IFN responses elicited by TLR2 ligands was contamination. To rule out such a possibility, macrophages from *TLR2^−/−^* and *MyD88^−/−^* mice were analyzed. Indeed, TLR2 ligands elicited no response in *TLR2^−/−^* and *MyD88^−/−^* macrophages (**Fig. 1A–F** and **Fig. S1**). We also analyzed macrophages from mice deficient in *IFN-β* or the type I interferon receptor (*IFNAR*). *IFN-β^−/−^* and *IFNAR^−/−^* macrophages were impaired in the induction of type I IFN-dependent genes IP-10, MX-2, iNOS and IL-6 but not in the pro-inflammatory cytokines TNF-α or IL-12 (**Fig. 1A, B, D, E** and **Fig. S1**). Interestingly, the impairment in *IFN-β^−/−^* macrophages was equivalent to that in *IFNAR^−/−^* macrophages (**Fig. 1B, E** **,** and **Fig. S1**). Since the IFNAR is a common receptor for all type I IFNs, this observation suggested that the type I IFN-dependent responses induced by such agonists was largely due to the activity of IFN-β and not the other isotypes like IFN-αs.

This prompted us to test for IFN-β induction. Quantitative real-time RT-PCR and ELISA assays demonstrated that all the TLR2 ligands tested could trigger the transcription and subsequent secretion of IFN-β in a TLR2-MyD88 dependent manner (**Fig. 1C and F**). Intriguingly, *IFNAR^−/−^* macrophage cultures were repeatedly found to contain more IFN-β (**Fig. 1F**). Such difference was however not reflected at the mRNA levels (**Fig. 1C**), suggesting that higher levels of IFN-β in *IFNAR^−/−^* cultures was due to lack of consumption via IFNAR. Taken as a whole, these results show that activation of the TLR2-MyD88 pathway leads to the production of IFN-β which in turn induces type I IFN-dependent responses.

**Figure 2:**
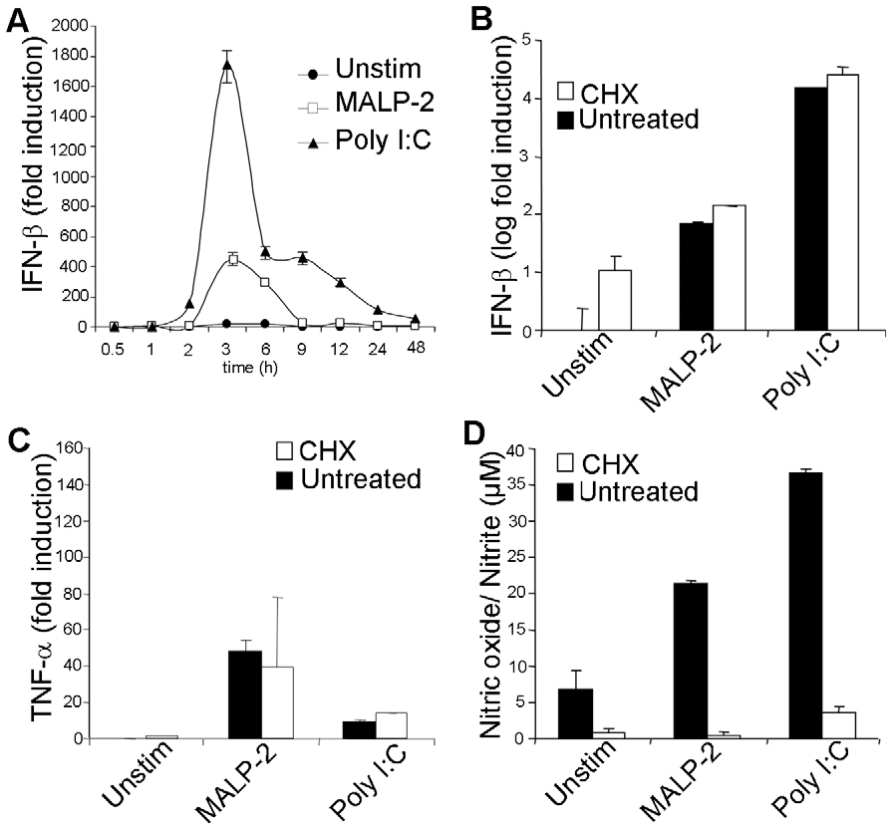
TLR2 activation induces IFN-β transcription independent of an auto/paracrine loop. In **A,** BMDMs were stimulated with either MALP-2 (1.5 µg/ml) or Poly I:C (5 µg/ml) for the indicated time periods then analyzed by quantitative real time RT-PCR for IFN-β mRNA. **B–D;** TLR2 driven IFN-β induction is independent of an auto/paracrine mechanisms. BMDMs pre-treated for 1 h with or without 0.5 µg/ml CHX were stimulated for 6 h with either MALP-2 (1.5 µg/ml) or Poly I:C (5 µg/ml) then analyzed by real-time RT-PCR for IFN-β (**B**, please note logarithmic scale) and TNF-α (**C**). Culture supernatants of cells stimulated in parallel for 24 hours in the presence of cycloheximide were analyzed for nitric oxide (**D**). Data are representative of three independent experiments (mean ± s.e.m).

These findings are contrary to several previous studies [14], [15], [16], [17]. However, recently, while this study was under consideration elsewhere, Barbalat and co-workers could show TLR2 - driven type I IFN responses but only toward viral and not bacterial ligands [18]. Whereas the explanation for the previous failures to detect TLR2-driven type I IFN responses is not very certain, several factors including sub-optimal activation of TLR2, low sensitivity of assays and/or measuring type I IFN response off peak period could have been responsible. For example low concentrations of TLR2 ligands and sub-optimal northern blotting and RT-PCR conditions detecting only very strong responses could account for some of the previous observations [14], [15], [17], [18]. For instance, it is well known, and also shown herein (Fig. 2A and Fig S2A), that TLR ligands induce IFN-β with a rapid kinetic with mRNA peaks between 1 and 6 hours post stimulation followed by a rapid decline to near basal level by 12 hour post stimulation. In the study by Barbalat and colleagues, the conclusions that bacterial TLR2 ligands do not induce type I IFNs were largely based on experiments measuring IFN-β transcript 12–24 hours–a time window not optimal for detection of IFN-β transcript. The fact that IFN-β induced by vaccinia virus could be detected at this time point is probably a reflection of the potential difference in the kinetics with which the purified TLR ligand and whole microorganisms (in this case vaccinia virus) interacts with the host cells. On the other hand, while Toshchakov and co-workers [17] also concluded that TLR2 ligands do not induce type I IFNs in bone marrow macrophages, a critical analysis of their data suggests otherwise. Albeit lower than that by LPS, the authors could detect TLR2 driven transcriptional upregulation of IFN-β and the IFN dependent genes iNOS (Figure 5a and 1 respectively of that paper). The inability to also detect clear response for the other type I IFN genes like IP-10 could be due to low sensitivity of assays probably optimal for the detection of stronger responses triggered via TLR4 and not TLR2. A similar argument could also account the PCR and northern blot data in the other previous studies [14], [15] . In short, several reasons could account for the poor detection of TLR2-driven type I IFNs responses in previous studies and the explanations may vary from one experiment/study to another. Equally noteworthy, as we have shown herein (**Fig. 1F**) the fact that secreted IFN-β is rapidly depleted from the system via IFNAR could probably have undermined its detection in other previous studies. This is especially so if assayed long after the peak of production.

### TLR2 induces IFN-β directly

**Figure 3:**
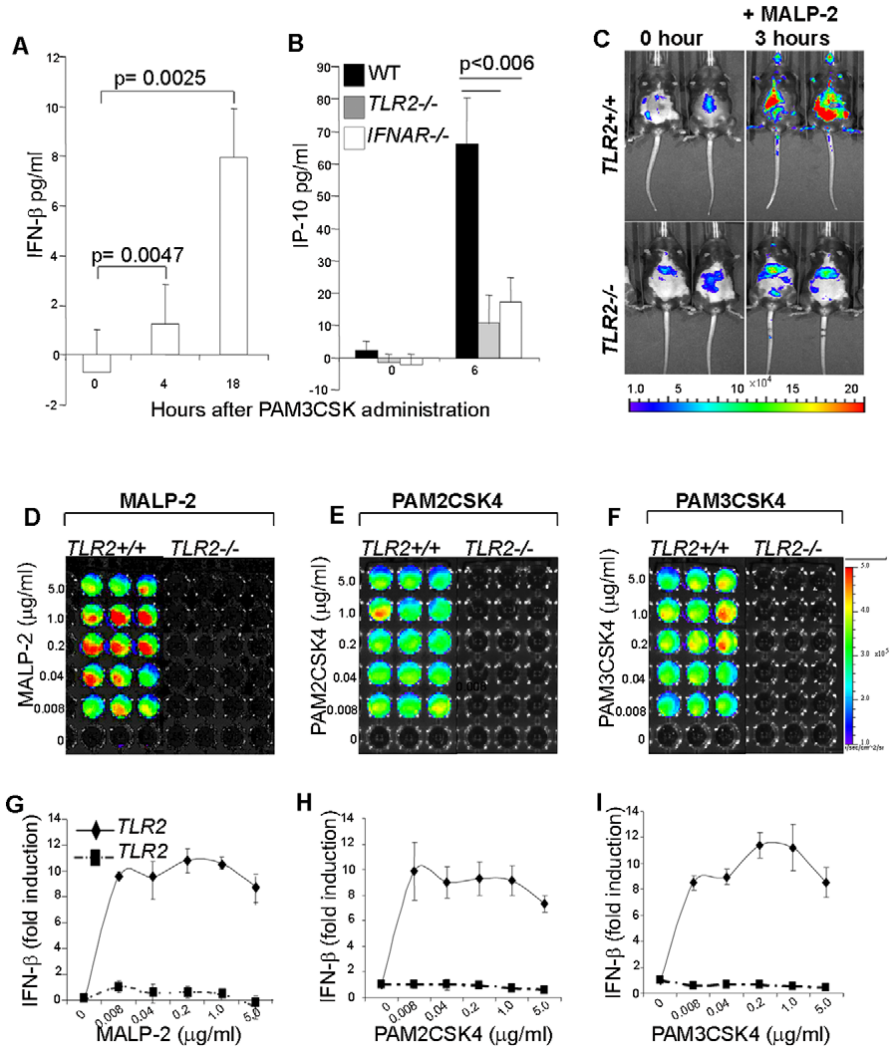
TLR2-driven IFN-β and type I IFN dependent responses *in vivo*. **A–B**; Five mice per group WT, TLR2*^−/−^* and IFNAR*^−/−^* were injected with control medium or PAM3CSK4 (100 µg/mouse) then sacrificed at different time points between 0–18 hours and their sera (**A**) or peritoneal exudates (**B**) analyzed by ELISA for IFN-β and IP-10 respectively Data representative of two independent experiment show ± s.e.m. **C**; Three mice per group of *TLR2^+/+^IFN-β^+/Δ-luc^* (*TLR2^+/+^*) and *TLR2^−/−^IFN-β^+/Δ-luc^* (*TLR2^−/−^*) mice were injected with MALP-2 (100 µg/mouse) and expression of luciferase was monitored for 24 h by luminescence imaging. **C** shows two representative mice out of a group of four mice before (0 hours) and after (3 hours) MALP-2 administration. Fold increase and kinetics of luciferase induction of corresponding mice are depicted in **Fig. S2A**. **D–I**; TLR2 mediated IFN-β induction in macrophages. Macrophages matured from bone marrows of *TLR2^+/+^IFN-β^+/Δ-luc^* or *TLR2^−/−^IFN-β^+/Δ-luc^* mice were stimulated with indicated concentrations of MALP-2 (**D**, **G**) or PAM2CSK4 (**E**, **H**) or PAM3CSK4 (**F**, **I**) for 4 hours and analyzed for luciferase expression. **D–F**; show representative luminescence images of macrophages while **G–I** indicate the fold increase in luciferase in corresponding cells. Luciferase expression is represented by a colour shift from blue to red. Similar results were obtained from three independent experiments (mean ± s.e.m). Note that the background response in reporter mice, mainly in the liver region, might be due to the liver's role in trapping and detoxifying noxious substances from the system. Such substances and the metabolic products thereof could account for the higher MALP-2 associated background in TLR2−/− mice.

We also tested whether TLR2 induces IFN-β directly as opposed to a delayed response via auto/paracrine mechanisms. To that end, first, we did a time course analysis by real-time RT-PCR, to determine the kinetic of IFN-β gene induction. TLR2 agonists induced IFN-β transcription with a rapid kinetic (peak at 2–3 h). This fast response was comparable to that by Poly:IC (TLR3 ligand) which is known to activate IFN-β directly (**Fig. 2A**). As depicted below, rapid IFN-β induction upon TLR2 activation was also evident *in vivo* (**Fig. 3A, C** and **Fig. S2A**). Nonetheless, to compellingly test whether IFN-β induction is independent of *de novo* protein synthesis, cells were stimulated with TLR2 and TLR3 agonists in the presence or absence of the protein synthesis inhibitor cycloheximide (CHX). In spite of complete inhibition of protein synthesis, as depicted by the absence of NO and IL-6 in supernatants (**Fig. 2D** and **data not shown**), TLR2 and TLR3 ligands still induced the transcription of IFN-β and TNF-α in the presence of CHX (**Fig. 2B–C**). Thus, much like TLR3, activation of TLR2 does induce both pro-inflammatory and IFN-β responses directly.

### TLR2-driven IFN-β induction *in vivo*

To investigate TLR2-driven type I IFN responses *in vivo*, WT, TLR2^−/−^ and IFNAR^−/−^ mice were inoculated with the TLR2 ligand PAM3CSK4 and then analyzed for secretion of IFN-β and type I IFN-dependent mediators. ELISA assay readily revealed an accumulation of IFN-β in the serum of *IFNAR^−/−^* but not TLR2^−/−^ or WT mice (**Fig. 3A** and **data not shown**). This is consistent with the *in vitro* data showing that upon TLR triggering, IFN-β is secreted which in turn binds to and is thus removed from the system via IFNAR. Indeed, further analysis revealed elevated levels of IP-10 and NO in WT but not in *TLR2^−/−^* or *IFNAR^−/−^* mice (**Fig. 3B** and **data not shown**).

To monitor the *in vivo* induction of IFN-β in more details, we constructed a transgenic IFN-β-luciferase reporter mouse (*Δβ-luc*)[19]. In these mice, the *IFN-β* gene was replaced by the firefly luciferase. For the present analysis, we used *Δβ-luc* heterozygous mice containing a functional *IFN-β* allele and a luciferase expression cassette (*TLR2^+/+^IFN-β^+/Δ-luc^*). For control, the *Δβ-luc* mice were crossed with *TLR2^−/−^* mice to generate *TLR2^−/−^IFN-β^+/Δ-luc^* mice. When challenged with MALP-2 or PAM3CSK4, *TLR2^+/+^IFN-β^+/Δ-luc^* mice responded with luminescence while mice lacking *TLR2* (**Fig. 3C** and **Fig. S2A**). Consistent with the rapid kinetics of IFN-β induction observed *in vitro* (**Fig. 2A**) the peak of the *in vivo* IFN-β response by such TLR2 ligands was also 3-4 hours post stimulation (**Fig. 3C** and **Fig. S2A**).

Over the years it had been suggested that only the TLR9 subfamily (TLR7, TLR8 and TLR9) could activate type I IFNs via MyD88. In fact, even the MyD88 pathways for IFN induction by such TLRs was believed to be functional only in plasmacytoid dendritic cells (pDCs) [20], [21]. However recently Mancuso and co-workers were able to demonstrate a role for TLR7-MyD88 dependent pathway of type I IFN induction in conventional DCs in response to group B streptococcus [22]. To investigate whether the TLR2-MyD88 pathway of IFN-β induction is also functional in other cell types, peritonial macrophages, bone marrow derived macrophages (BMDM) and dendritic cells (BMDCs) were also tested in an *in vitro* stimulation assay with graded concentrations of TLR2 and TLR4 agonists. TLR2 agonists were found to induce luciferase in both, macrophages and DCs from *TLR2^+/+^* but not *TLR2^−/−^* mice (**Fig. 3D–I** and **Fig. S2B and data not shown**). In contrast, LPS induced responses in both, *TLR2^+/+^* and *TLR2^−/−^*, cells (**Fig. S2C&D**). Furthermore, LPS stimulation depicted stronger responses as compared to TLR2 which is consistent with the activation of the TRIF pathway associated with very strong type I IFN induction.

In a recently study, Barbalat and co-workers also proposed TLR2 - dependent type I IFN production only in response to viral but not toward bacterial ligands. The authors also proposed that such responses were only feasible in inflammatory monocytes [18]. The functional role of inflammatory monocytes in the study in question was based on depletion of cells expressing CD11b - a molecule generally expressed on several cell types including monocytes, macrophages, granulocytes, and dendritic cells. This raises the possibility that such responses were in fact the sum of several cell types and not just inflammatory monocytes. Whichever the case and in view of our findings it can safely be concluded that TLR2 pathway of IFN-β induction is functional in several host cell types and that this pathway is not a preserve of viral activation but rather a consequence of TLR2 activation by various ligands including those from bacteria, mycoplasma and possibly fungi and protozoa.

### TLR2 induces type I IFN-β via IRF1 and IRF7

**Figure 4:**
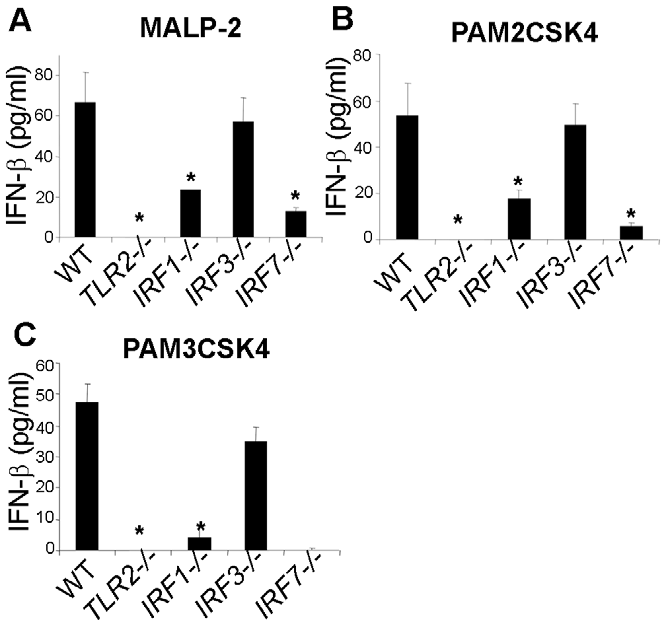
TLR2 activation induces type I IFN-β via IRF1 and IRF7. **A–C,** WT, *TLR2* ***^−/−^***, *IRF1* ***^−/−^***, *IRF3* ***^−/−^***, and *IRF7* ***^−/−^*** BMDMs were stimulated with ligands for TLR2/6 (1.5 µg/ml MALP-2 or PAM2CSK4) or TLR2/1 (1.5 µg/ml PAM3CSK4). After 10 hours, supernatants were analyzed for IFN-β by ELISA. Similar data represent an average of two independent experiments (mean ± s.e.m, *: p<0.05).

The transcription of IFN-α/β is primarily controlled by the IRF family of transcription factors. To elucidate the IRFs downstream of the TLR2-MyD88 pathway of IFN-β induction, we investigated the role of IRF1, IRF3 and IRF7. These transcription factors have been implicated as positive regulators of the transcription of type I IFN genes [23]. Macrophages from *IRF1^−/−^* and *IRF7^−/−^* mice were highly diminished in IFN-β production following stimulation with TLR2 ligands, while *IRF3^−/−^* macrophages had a normal response (**Fig. 4A–C**). Based on the present data, TLR2 activation induces type I IFN responses via MyD88-IRF1 and -IRF7 pathways. These findings suggest that the TLR2-driven pathway of type I IFN induction is in a way similar to that by the TLR9 subfamily of TLRs also shown to trigger MyD88-IRF1 and -IRF7 pathways [22], [24], [25], [26], [27].

### TLR2 induces IFN-β from endolysosomes

**Figure 5:**
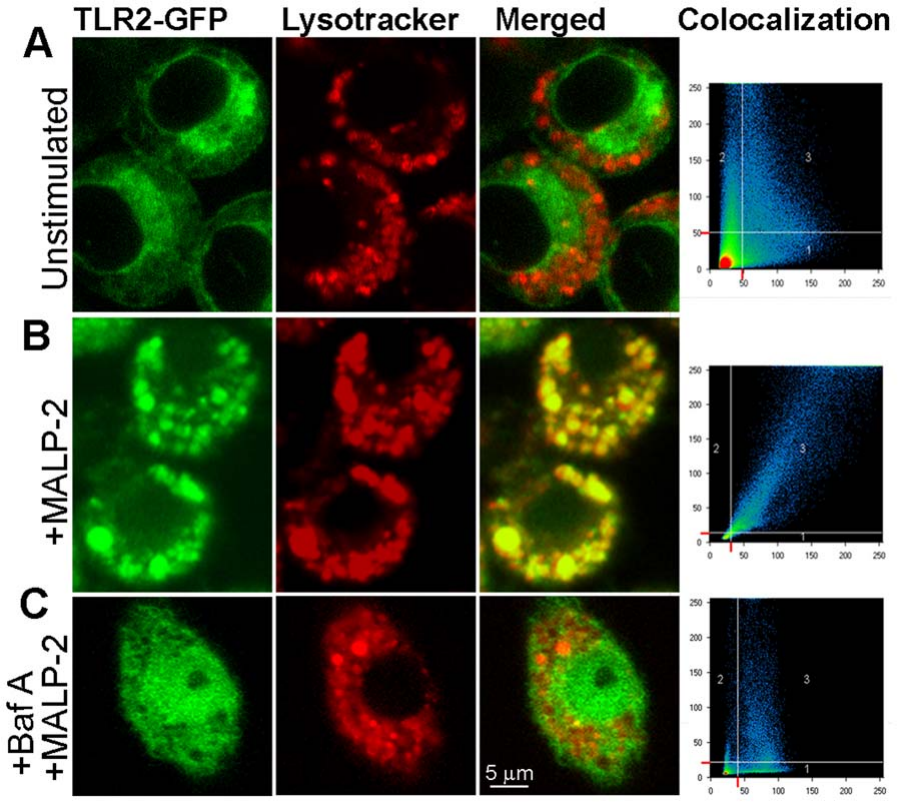
Ligand induced internalization and trafficking of TLR2 into endolysosomal compartments. RAW 264.7 macrophages transfected with hTLR2-GFP were labelled with lysotracker then left untreated (**A**) or stimulated with 1 µg/ml of MALP-2 and imaged by confocal microscopy after 3 h (**B**). Panel **C** shows cells first labelled with lysotracker then incubated for 1 hour with 50 µM Bafilomycin A before stimulation with MALP-2. The co-localization fluorographs displayed in the right panels show the intensities and scatter pattern of all pixels within the merged images. Pixels with mostly one fluorescent component are placed along the axes 1 and 2 while the pixels with equal fluorescence intensity from both components (due to co-localization) are placed along the diagonal. Axis 1 and 2 show the green (TLR2-GFP) and red (Lysotracker) fluorescence intensities on an arbitrary scale of 0 to 250.

One of the emerging themes in the induction of type I IFNs is the intracellular localization of receptors. Potent inducers of type I IFNs like TLR3, TLR7/8, TLR9, RIG-I, MDA5, DAI, are all intracellular receptors that sense nucleic acids located in the endosome or cytosol [5], [8]. Using confocal microscopy, we first studied the sub-cellular localization of TLR2-GFP in transiently transfected RAW 264.7 macrophages. Consistent with previous observations [28], in unstimulated cells, TLR2-GFP was found to be localized either at the plasma membrane or in the perinuclear region (most likely in ER and trans-golgi networks) and hardly co-localized with the endolysosomal markers lysotracker or dextran (**Fig. 5A** and **Fig. S3A**). However by 3 h after MALP-2 stimulation, TLR2 was found enriched in clusters which co-localized with lysotracker or Dextran (**Fig. 5B** and **Fig. S3B).** Such co-localization was however severely impaired when the endocytic maturation pathway was blocked using Bafilomycin A (BafA), an inhibitor of the endosomal proton pump **(** **Fig. 5C** and **Fig. S4C)**. These observations suggest that TLR2 activates endocytic internalization and endosome maturation, as proposed previously [29], [30], [31].

**Figure 6:**
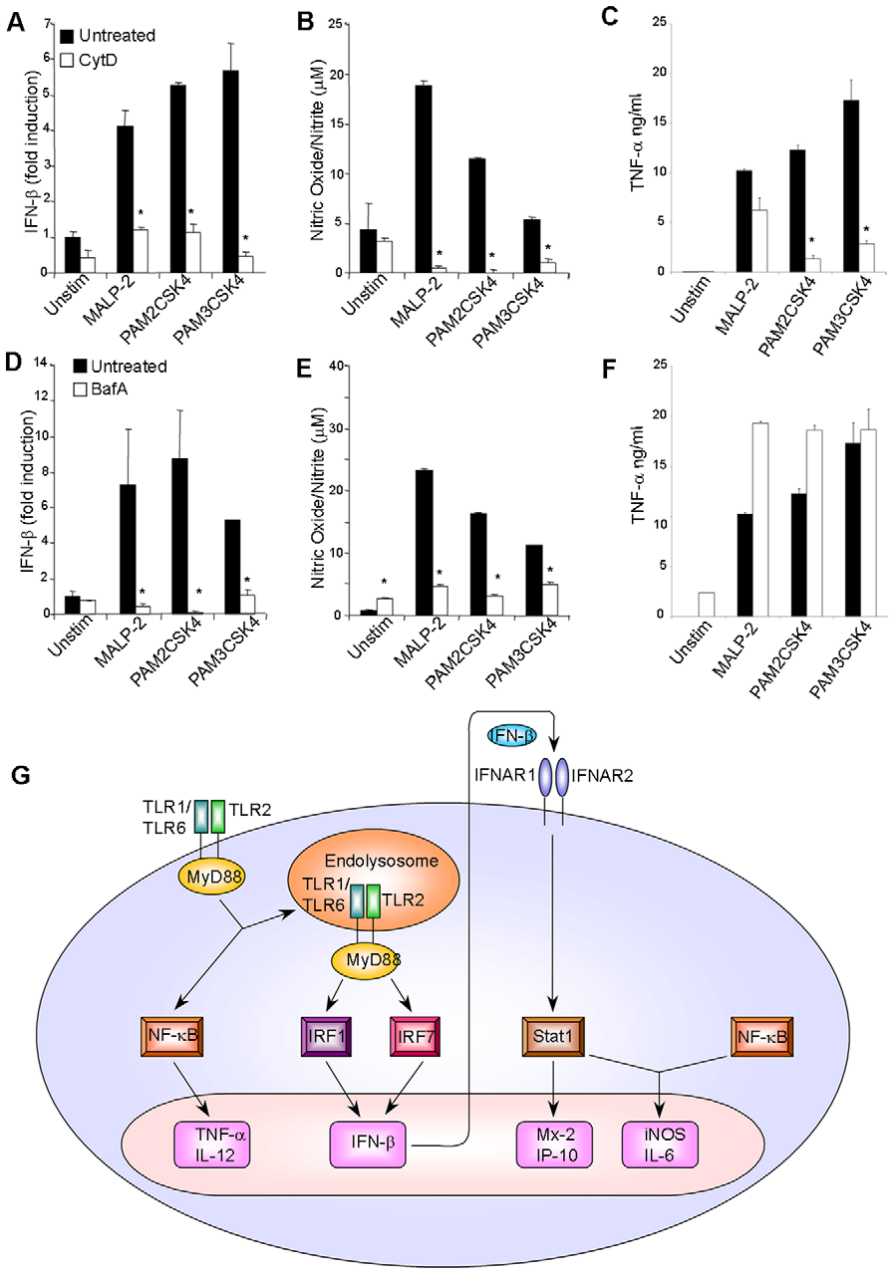
Blockers of receptor internalization and endosome maturation pathways abrogate TLR2 driven type I IFN responses. BMDM, untreated or pre-treated with either 25 µM Cytochalasin D (A–C) or 50 µM Bafilomycin A (D–F) for 1 hour, were stimulated with 1.5 µg/ml of the indicated TLR2 ligands. After 6 h cell pellets were analyzed by quantitative real-time RT-PCR for IFN-β (A and D). Supernatants of cells stimulated in parallel for 24 hours were also analyzed for Nitric Oxide (Nitrite) by Griess reaction (B and E) or for TNF-α. Data represent similar results from three independent experiments (mean ± s.e.m, *: p<0.05). G; Model for TLR2 signaling. Upon ligand engagement at the plasma membrane TLR2 activates a MyD88-NF-κB pathway leading to the induction of pro-inflammatory cytokines like TNF-α and IL-12. The initial trigger at the plasma membrane also induces endocytic internalization and transportation of the TLR2 complex into endolysosomal compartments from where it activates MyD88-IRF1/IRF7 pathways of IFN-β induction. Once produced, IFN-β in turn activates STAT1 which alone or in synergy with other transcription factors like NF-κB activates the transcription of IFN-inducible genes; Mx-2, IP-10, iNOS, IL-6.

To investigate a potential link between the translocation of TLR2 into endolysosomal compartments and the induction of type I IFN responses, macrophages were stimulated with TLR2 ligands in the presence of Cytochalasin D (CytD). CytD blocks endocytic internalization by inhibiting actin polymerization. CytD almost completely inhibited the induction of IFN-β and type I IFN-inducible genes (IP-10, and iNOS) by TLR2 ligands (**Fig. 6A–C**). In addition to blocking receptor internalization and vesicular trafficking, CytD may also interfere with other signalling processes. Therefore, we resorted to a more specific inhibitor of the endocytic maturation pathway: Bafilomycin A (BafA). BafA inhibits the endosomal proton pump hence endosome acidification and subsequent fusion with lysosomes. BafA severely inhibited the TLR2 driven IFN-β and type I IFN dependent responses but not the pro-inflammatory responses (**Fig. 6D–F**). Similar results were obtained with Dynasore (Dyn), an inhibitor of the endocytic effector protein dynamin (**Fig. S4**). Collectively, these data suggest that TLR2 induces pro-inflammatory cytokines like TNF-α from the plasma membrane while IFN-β is triggered from endolysosomal compartments after receptor internalization.

## Discussion

So far, the signalling model for the TLR2 subfamily of TLRs has been based on the dogma that these receptors induce pro-inflammatory cytokines but not type I interferons upon activation by bacterial ligands. Using different *in vitro* and *in vivo* approaches, we now show that in addition to pro-inflammatory cytokines, TLR2 heterodimers also elicit interferon-β and hence type I interferon dependent responses. Interestingly, the induction of pro-inflammatory responses and type I interferons by TLR2 ligands occur from distinct sub-cellular sites: the plasma membrane and endolysosomal compartments, respectively.

As schematically illustrated in **Fig. 6G**, our results suggest a new model for TLR2 signalling. We propose that analogous to that for TLR4, TLR2 activation at the plasma membrane first induces the MyD88-NF-κB-dependent pathway. This activates not only the transcription of genes encoding pro-inflammatory cytokines but also induces endocytic internalization and trafficking of the TLR2 complex into endolysosomal compartments, where it additionally activates MyD88-dependent IRF1 and IRF7 pathways for IFN-β induction. Consequently, as we have shown, pharmacological blockers of receptor internalization and endosome maturation are able to inhibit the induction of type I interferons but not pro-inflammatory cytokines upon TLR2.

Type I IFNs support the transcription of several mediators that promote protection against microbes. Some of the type I IFN-inducible mediators such as IP-10 and NO, herein shown to be induced in response to TLR2 activation play an important role not only in the activation and recruitment of inflammatory cells to sites of infection but also have direct anti-microbial effects. For example, TLR2-driven NO production, is known to be critical for protective immunity against *Toxoplasma gondii* [32]. Similarly, NO production, also a critical protective component against mycoplasmas [33], is known to be largely due to TLR2 activation by MALP-2 or its homologs [34], [35].

In a recent study, Barabalat and co-workers reported a role for TLR2 in the recognition of vaccinia virus [18]. However since such studies employed UV irradiated virus particles, incapable of activating cytosolic signalling pathways, whether type I IFN responses induced by vaccinia virus are mainly due to TLR2 activation as proposed [18], as opposed to the TLR independent cytosolic pathways as reported by Zhu and others [36] remains to be resolved. Nonetheless, together with the present findings it is clear that although so far ignored, TLR2–driven pathway of type I IFN induction is a crucial component of host defences against a variety of microbes.

The mechanistic explanation for the induction of type I IFNs from endolysosomal compartments by TLR2 is not clear at the present. However, we are tempted to speculate that such intracellular compartments could serve as docking sites on which key signalling molecules involved in type I IFN induction are marshalled to a sufficient concentration to mediate downstream signalling processes. We also infer that the acidic environment in endolysosomal compartments probably facilitates conformational changes in TLRs that initiate interactions with downstream adaptors, kinases and IRFs. In line with this hypothesis, DNA structures that target different endocytic compartments are known to induce distinct biological responses [21], [37], [38], and have been shown to trigger different conformational changes in TLR9 [39]. A more recent study showing that proteolytic processing of TLR7 and TLR9 by endolysosomal proteases is a prerequisite for their signalling [40] is also consistent with the above idea. Additional studies are required to provide more mechanistic insights into how intracellular localization and an acidic environment facilitate the induction of type I IFNs by TLRs and other PRRs.

Finally, our results have clinical implications. Although the TLR triggering does mediate protective host responses against microbes, excessive production of pro-inflammatory cytokines and type I IFNs due to such activation is known to inflict severe self-injury. Thus the discovery that pro-inflammatory and type I IFN responses are induced from distinct sub-cellular sites and that these two responses can be uncoupled by properly manipulating the endosome maturation pathway suggest new avenues for potential therapeutic intervention against inflammations.

## Materials and Methods

### Ethics statement

All mice were maintained under pathogen free conditions and experiments were approved and carried out according to the guidelines set out by LAVES (Niedersaechsisches Landesamt für Verbraucherschutz und Lebensmittelsicherheit).

### Mice

C57BL/6 mice were purchased from Harlan- Germany. *IRF1* ^−/−^ [41], *IFNAR^−/−^* [42] and *IFN-β^−/−^* [43] mice were bred in our animal facility. *TLR2^−/−^* [44] and *MyD88* ^−/−^ [45] mice, originally generated in Dr. S Akira's laboratory, were obtained from MPI-Berlin. *IRF3* ^−/−^ [46], and *IRF7* ^−/−^ [25] mice were obtained from Dr. T. Decker (University of Vienna, Austria) with permission from Dr. T. Taniguchi (University Tokyo, Japan). The transgenic IFN-β-luciferase reporter mice (*Δβ-luc*) [19] were generated by replacing IFN-β with the firefly luciferase using the standard homologous recombination protocol. The *Δβ-luc* mice were then backcrossed to *TLR2^−/−^* mice [44] to generate *TLR2^-/-^IFN-β^+/Δ-luc^* and *TLR2^+/+^IFN-β^+/Δ-luc^* mice.

### Reagents

MALP-2 was a kind gift from Dr. Werner Tegge, HZI. PAM2CSK4, PAM3CSK4, and LPS *Salmonella minnesota* were from Invivogen. Poly I:C was from Amersham Biosciences. Cytochalasin D was purchased from FLUKA, Cycloheximide, Bafilomycin A and Dynasore were purchased from Sigma.

### Cells

Bone marrow derived macrophages were cultured for 7 days in the presence of M-CSF and bone marrow derived dendritic cells were cultured for 10 days in the presence of GM-CSF and IL-4 according to standard protocols.

### Cell transfection and confocal imaging

hTLR2-GFP was bought from Invivogen. RAW 264.7 cells were transiently transfected using MicroPorator (PM-100) according to the manufacturer's instructions. hTLR2-GFP transfected cells were loaded with 1 µM of Lysotracker Red DND-99 or Dextran-Alexa Fluor 647 (both from Invitrogen) for 30 minutes, washed, then incubated with Bafilomycin A (in some cases) before stimulation with TLR2 ligands. Confocal imaging was done as described previously [47].

### Cytokine Assays

Supernatants were analyzed by ELISA using rat anti-mouse IL-12p70 and rat anti-mouse IL-6 antibodies together with the respective recombinant mouse IL-6 and IL-12 standards from BD Pharmingen. The IFN-β ELISA kit was purchased from PBL Biomedical Laboratories and the IP-10 ELISA was from Bender MedSystems. Both were performed according to the manufacturer's instructions. Nitric oxide was detected using the Griess reagent kit (Promega).

### *In vivo* luciferase assays

Mice were injected intraperitoneally with TLR2 ligands. At different time points, mice were anaesthetized with isoflurane using the XGI-8 gas anaesthesia system (Caliper). Prior to image acquisition 3 mg of luciferin (Caliper) dissolved in 100 ml PBS was injected intraperitoneally. Images were obtained at consecutive time points thereafter using the IVIS-200 system (Caliper) according to instructions of the manufacturer. The software Living image 3.0 (Caliper) was used for image analysis and quantification of emission intensities.

### *In-vitro* luciferase assays

Cells were stimulated with TLR2 agonists for 4 hours. The cells were lysed in Reporter Lysis Buffer (Promega). 5 µl of cell lysate was added to 25 µl of Luciferase Assay Reagent II (Promega) and Images were obtained using the IVIS-200 system (Caliper) Luminescence was quantified as described above.

### Statistical analysis

Data in the text and figures are expressed as the mean +/− SD. Statistical comparisons were carried out using the Student's *t*-test. *P* values less than 0.05 were considered significant.

### PCR

RNA extraction was performed with the Quiagen Rneasy Mini Kit according to the manufacturer's instructions. RT-PCR was performed with the GoTaq Flexi DNA Polymerase from Promega and Real time PCR was carried out using Power SYBR Green PCR Master Mix from Applied Biosystems with the following primers (operon):

TNF-α sense (5′-TCTCATCAGTTCTATGGCCC-3′),

TNF-α antisense (5′-GGGAGTAGACAAGCTACAAC-3′),

IP-10 sense (5′-GATGACGGGCCAGTGAGAATGAG-3′),

IP-10 antisense (5′-CTGGGTAAAGGGGAGTGATGGAGA-3′),

Mx-2 sense (5′-GAGAATGTCGCCTATTCACCAGGCTC-3′),

Mx-2 antisense (5′-GCCATGGATGAGGTCTGTAAATCTTGGG-3′),

IFN-β sense (5′-CATCAACTATAAGCAGCTCCA-3′),

IFN-β antisense (5′-TTCAAGTGGAGAGCAGTTGAG-3′),

RPS9 sense (5′-CTGGACGAGGGCAAGATGAAGC-3′)

and RPS9 antisense (5′-TGACGTTGGCGGATGAGCACA-3′).

## Supporting Information

Figure S1 TLR2 activation induces type I IFN dependent responses via MyD88. WT, *TLR2^−/−^*, *MyD88^−/−^*, *IFN-β^−/−^* and *IFNAR1^−/−^* macrophages were stimulated (or unstimulated) with 1.5 µg/ml of MALP-2, PAM2CSK4 or PAM3CSK4. After 24 hours respective culture supernatants were analyzed for IL-6 by ELISA (A) or Nitric oxide/Nitrite by Griess reaction (B). Data are representative of more than four independent experiments (mean ± s.e.m.).
(1.41 MB TIF)

Figure S2 *In vivo* and *in vitro* analysis of IFN-β induction using the Luciferase reporter system. A shows the kinetics of IFN-β induction in *TLR2^+/+^IFN-β^+/Δβ-luc^* (*TLR2^+/+^*) and *TLR2^−/−^IFN-β^+/Δβ-luc^* (*TLR2^−/−^*) mice injected with MALP-2 (*: p<0.05). The corresponding luminescence images are depicted in Fig. 3C–D. Dendritic cells (BMDCs) or macrophages (BMDMs) derived from *TLR2^+/+^IFN-β^+/Δβ-luc^* (*TLR2^+/+^*) and *TLR2^−/−^IFN-β^+/Δβ-luc^* (*TLR2^−/−^*) mice were stimulated with indicated concentrations of MALP-2 or LPS for 4 hours and analyzed for luciferase expression. B shows quantification of fold increase in luciferase expression (i.e., IFN-β induction) in BMDCs (mean ± s.e.m.). C show representative luminescence images of macrophages while D indicates the fold increase in luciferase in corresponding cells (mean ± s.e.m.). Note that the luminescence colour scale bar for LPS and MALP-2 are different.
(3.10 MB TIF)

Figure S3 Ligand induced internalization and trafficking of TLR2 into endolysosomal compartments. RAW 264.7 macrophages transfected with hTLR2-GFP were loaded with fluorescent Dextran-Alexa Fluor 647, then left untreated (A) or stimulated with 1.5 µg/ml of MALP-2 and imaged by confocal microscopy after 3 hours (B). Panel C shows cells first labelled with Detran-Alexa and then pre-incubated for 1 h with 50 µM Bafilomycin A before stimulation with MALP-2 for 3 hours.
(1.38 MB TIF)

Figure S4 Dynasore, an inhibitor of dynamin 1, abrogates TLR2-driven IFN-β induction. BMDM from *TLR2^+/+^IFN-β^+/Δβ-luc^* mice pre-incubated for 1 h with indicated concentrations of Dynasore were stimulated with or without 1.5 µg/ml of MALP-2 for 4 h and analyzed for luciferase expression. A shows representative luminescence images of stimulated macrophages while B indicates the corresponding fold increase in luciferase activity. Luciferase expression is represented by a colour shift from blue to red. Data represent similar results from two independent experiments (mean ± s.e.m.). *: p<0.05 indicate statistical significance in the deference between Dynasore treated and untreated cells that were stimulated with MALP-2.
(3.00 MB TIF)
