# Supplementary figures and images for: Murine Toll-Like Receptor 2 Activation Induces Type I Interferon Responses from Endolysosomal Compartments
Source: PLoS One. 2010 Apr 20;5(4):e10250. doi: 10.1371/journal.pone.0010250 (PMC2857745; doi:10.1371/journal.pone.0010250)

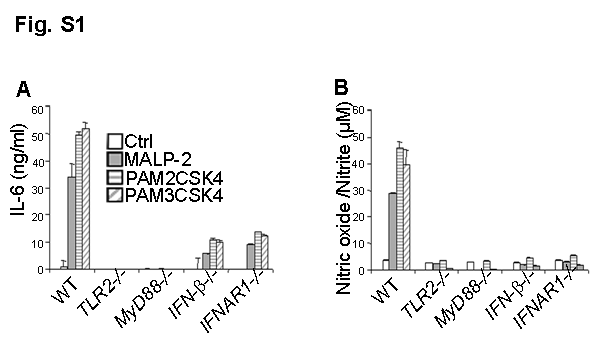

Supplement: Figure S1 — TLR2 activation induces type I IFN dependent responses via MyD88. WT, TLR2−/−, MyD88−/−, IFN-β−/− and IFNAR1−/− macrophages were stimulated (or unstimulated) with 1.5 µg/ml of MALP-2, PAM2CSK4 or PAM3CSK4. After 24 hours respective culture supernatants were analyzed for IL-6 by ELISA (A) or Nitric oxide/Nitrite by Griess reaction (B). Data are representative of more than four independent experiments (mean ± s.e.m.). (1.41 MB TIF) [file pone.0010250.s001.tif]

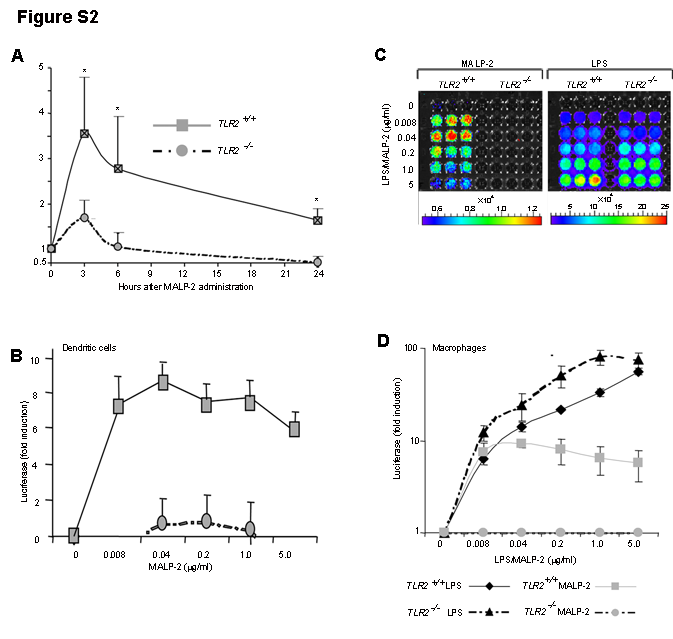

Supplement: Figure S2 — In vivo and in vitro analysis of IFN-β induction using the Luciferase reporter system. A shows the kinetics of IFN-β induction in TLR2+/+IFN-β+/Δβ-luc (TLR2+/+) and TLR2−/−IFN-β+/Δβ-luc (TLR2−/−) mice injected with MALP-2 (*: p<0.05). The corresponding luminescence images are depicted in Fig. 3C–D. Dendritic cells (BMDCs) or macrophages (BMDMs) derived from TLR2+/+IFN-β+/Δβ-luc (TLR2+/+) and TLR2−/−IFN-β+/Δβ-luc (TLR2−/−) mice were stimulated with indicated concentrations of MALP-2 or LPS for 4 hours and analyzed for luciferase expression. B shows quantification of fold increase in luciferase expression (i.e., IFN-β induction) in BMDCs (mean ± s.e.m.). C show representative luminescence images of macrophages while D indicates the fold increase in luciferase in corresponding cells (mean ± s.e.m.). Note that the luminescence colour scale bar for LPS and MALP-2 are different. (3.10 MB TIF) [file pone.0010250.s002.tif]

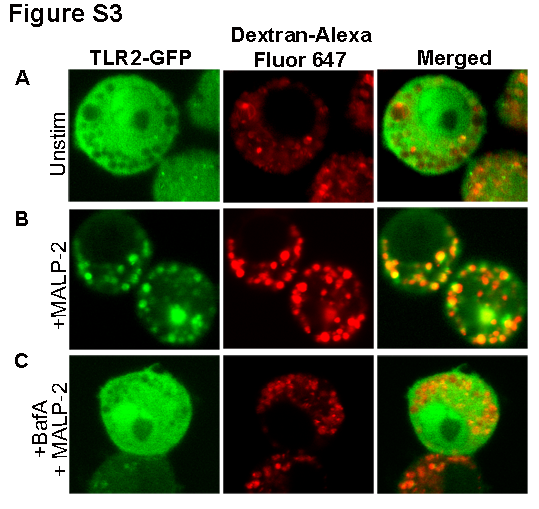

Supplement: Figure S3 — Ligand induced internalization and trafficking of TLR2 into endolysosomal compartments. RAW 264.7 macrophages transfected with hTLR2-GFP were loaded with fluorescent Dextran-Alexa Fluor 647, then left untreated (A) or stimulated with 1.5 µg/ml of MALP-2 and imaged by confocal microscopy after 3 hours (B). Panel C shows cells first labelled with Detran-Alexa and then pre-incubated for 1 h with 50 µM Bafilomycin A before stimulation with MALP-2 for 3 hours. (1.38 MB TIF) [file pone.0010250.s003.tif]

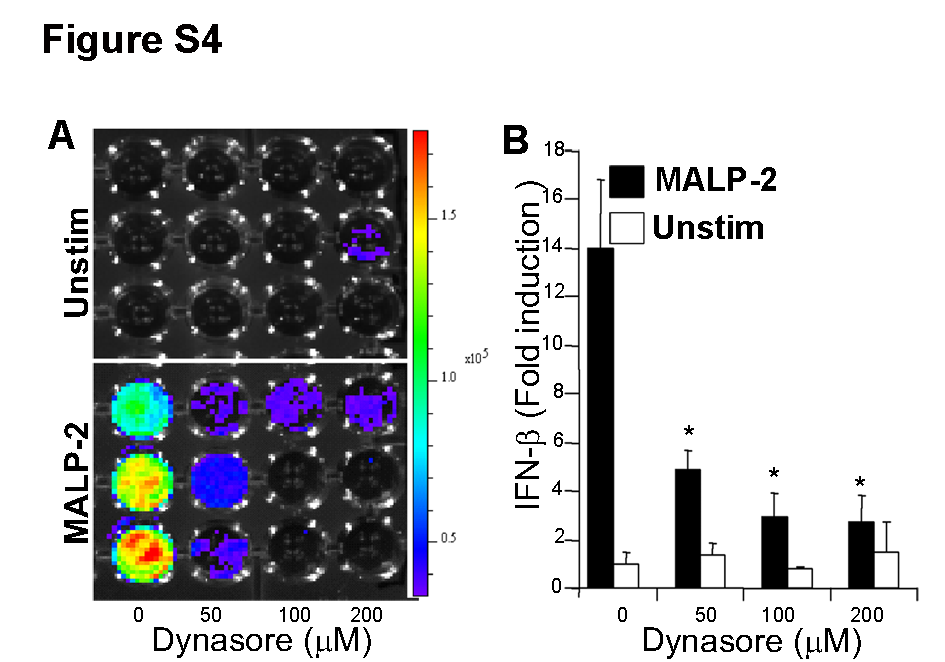

Supplement: Figure S4 — Dynasore, an inhibitor of dynamin 1, abrogates TLR2-driven IFN-β induction. BMDM from TLR2+/+IFN-β+/Δβ-luc mice pre-incubated for 1 h with indicated concentrations of Dynasore were stimulated with or without 1.5 µg/ml of MALP-2 for 4 h and analyzed for luciferase expression. A shows representative luminescence images of stimulated macrophages while B indicates the corresponding fold increase in luciferase activity. Luciferase expression is represented by a colour shift from blue to red. Data represent similar results from two independent experiments (mean ± s.e.m.). *: p<0.05 indicate statistical significance in the deference between Dynasore treated and untreated cells that were stimulated with MALP-2. (3.00 MB TIF) [file pone.0010250.s004.tif]
